# CT radiomics facilitates more accurate diagnosis of COVID-19 pneumonia: compared with CO-RADS

**DOI:** 10.1186/s12967-020-02692-3

**Published:** 2021-01-07

**Authors:** Huanhuan Liu, Hua Ren, Zengbin Wu, He Xu, Shuhai Zhang, Jinning Li, Liang Hou, Runmin Chi, Hui Zheng, Yanhong Chen, Shaofeng Duan, Huimin Li, Zongyu Xie, Dengbin Wang

**Affiliations:** 1 Department of Radiology, Xinhua Hospital, Shanghai Jiao Tong University School of Medicine, No. 1665 Kongjiang Road, Shanghai, 200092 China; 2 Department of Emergency, Xinhua Hospital, Shanghai Jiao Tong University School of Medicine, Shanghai, 200092 China; 3 Department of Radiology, The First Affiliated Hospital of Bengbu Medical College, No. 287, Changhuai Road, Bengbu, 233004 Anhui China; 4 GE Healthcare, Shanghai, 210000 China

**Keywords:** COVID-19, Computed tomography, Pneumonia, Radiomics, Machine learning

## Abstract

**Background:**

Limited data was available for rapid and accurate detection of COVID-19 using CT-based machine learning model. This study aimed to investigate the value of chest CT radiomics for diagnosing COVID-19 pneumonia compared with clinical model and COVID-19 reporting and data system (CO-RADS), and develop an open-source diagnostic tool with the constructed radiomics model.

**Methods:**

This study enrolled 115 laboratory-confirmed COVID-19 and 435 non-COVID-19 pneumonia patients (training dataset, n = 379; validation dataset, n = 131; testing dataset, n = 40). Key radiomics features extracted from chest CT images were selected to build a radiomics signature using least absolute shrinkage and selection operator (LASSO) regression. Clinical and clinico-radiomics combined models were constructed. The combined model was further validated in the viral pneumonia cohort, and compared with performance of two radiologists using CO-RADS. The diagnostic performance was assessed by receiver operating characteristics curve (ROC) analysis, calibration curve, and decision curve analysis (DCA).

**Results:**

Eight radiomics features and 5 clinical variables were selected to construct the combined radiomics model, which outperformed the clinical model in diagnosing COVID-19 pneumonia with an area under the ROC (AUC) of 0.98 and good calibration in the validation cohort. The combined model also performed better in distinguishing COVID-19 from other viral pneumonia with an AUC of 0.93 compared with 0.75 (*P* = 0.03) for clinical model, and 0.69 (*P* = 0.008) or 0.82 (*P* = 0.15) for two trained radiologists using CO-RADS. The sensitivity and specificity of the combined model can be achieved to 0.85 and 0.90. The DCA confirmed the clinical utility of the combined model. An easy-to-use open-source diagnostic tool was developed using the combined model.

**Conclusions:**

The combined radiomics model outperformed clinical model and CO-RADS for diagnosing COVID-19 pneumonia, which can facilitate more rapid and accurate detection.

## Background

The ongoing pandemic of coronavirus disease 2019 (COVID-19) caused by a novel coronavirus “severe acute respiratory syndrome coronavirus 2” (SARS-CoV-2) has become a global threat [1, 2]. The high contagion of SARS-CoV-2 and the virulence to cause severe illness involving multiple organs has caused many countries into a dilemma for screening, diagnosing, and treatment with limited healthcare resources. As of September 5, a total of 26,654,344 worldwide confirmed cases and 875,400 deaths have been reported [3], and the numbers continue to grow. The nucleic acid test using reverse-transcription polymerase chain reaction (RT-PCR) for SARS-CoV-2 was regarded as the diagnostic gold standard but with various sensitivities ranging from 59 to 71% depending on viral load and test sample quality [4, 5]. Furthermore, the lengthy turnaround times for final diagnosis and shortage of RT-PCR kit will delay the treatment, which contributes to the dilemma.

Chest CT imaging is a widely available, time-saving, and non-invasive approach for detecting COVID-19 pneumonia. Previous studies revealed that chest CT could serve as an efficient tool for diagnosing COVID-19 pneumonia with high sensitivity and monitoring disease course [4, 6–8]. Recently, a multinational consensus statement from the Fleischner Society also declared that CT scanning can be a major method if symptoms worsen or there is a situation short of RT-PCR kit [9]. CT features including peripherally distributed ground-glass opacity (GGO), GGO with consolidation and/or reticulation were considered as typical imaging characteristics [6]. However, COVID-19 pneumonia shared similar imaging features with pneumonia caused by other pathogens, especially other viral pneumonia. The specificity was relatively low when compared to RT-PCR results [4], which meant CT could not fully exclude COVID-19 infection for suspected patients. Quarantine for those with final COVID-19 negative RT-PCR results increased stress on limited healthcare resources. As for distinguishing COVID-19 from other viral pneumonia on chest CT, high specificities but moderate sensitivities were reported among different international radiologists [10]. To facilitate the evaluation of COVID-19 pneumonia, a standardized assessment scheme for pulmonary involvement of COVID-19 named CO-RADS (COVID-19 reporting and data system) was developed to estimate the risk [11, 12]. The subjective CO-RADS classification demonstrated high discriminatory power but moderate to substantial agreement among observers. Hence, more measures should be taken for more rapid and accurate diagnosis of COVID-19 to combat the current pandemic.

Radiomics, a non-invasive machine learning technology, involved high-throughput extraction of a large number of quantitative features from medical images, thereby converting image data into high-dimensional data to objectively and quantitatively describe the characteristics of lesions that may not be perceptible by the naked eye. The potential benefits of radiomics had been highlighted in improving diagnostic, prognostic, and predictive accuracy for cancers such as lung cancer, rectal cancer, etc. as well as other non-neoplastic diseases [13–16]. To date, there are limited data about the value of chest CT-based radiomics in rapidly and accurately detecting COVID-19 pneumonia.

In the present study, we aimed to develop and validate a combined radiomics model including clinical characteristics and the radiomics signature for distinguishing COVID-19 from pneumonia with other etiologies by using real-world data during the COVID-19 outbreak period in China. Additionally, the predictive performance of the clinico-radiomics combined model was compared with the clinical model and CO-RADS grading approach by recruiting an independent viral pneumonia cohort.

## Materials and methods

### Patients

**Fig. 1:**
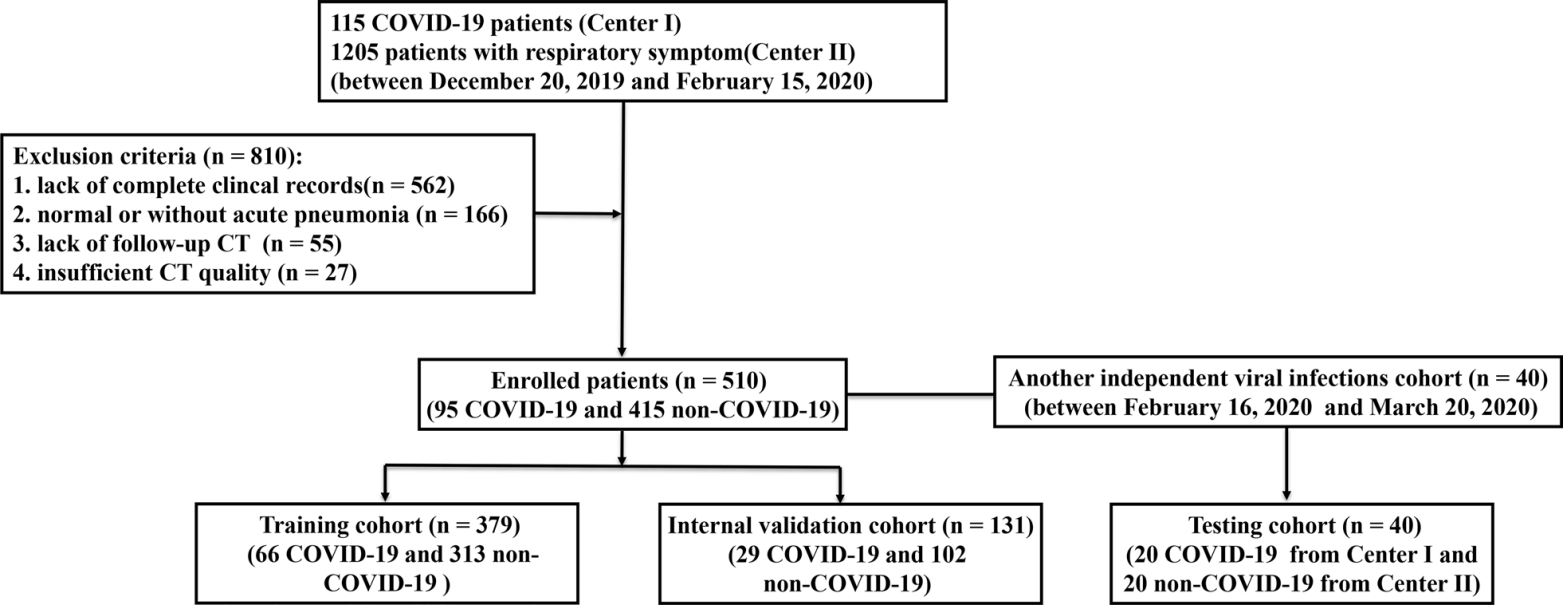
The workflow of this study

This study was approved by the Institutional Ethics Committee of Xinhua Hospital affiliated to Shanghai Jiao Tong University School of Medicine (No. XHEC-D-2020-090). The patient informed consent requirement was waived for this retrospective study using de-identified data. Clinical and non-contrast chest CT data of consecutive 115 patients with COVID-19 confirmed by RT-PCR from Bengbu City, Anhui Province (center I) as well as 1205 patients with respiratory symptoms from Xinhua Hospital (center II) were reviewed during the COVID-19 outbreak from December 20, 2019 to February 15, 2020. Patients with common pathogen confirmation and disease improvement on follow-up CT after treatment were grouped as non-COVID-19 pneumonia patients. The exclusion criteria were as follows: (a) lack of complete clinical records (blood test or pathogen confirmation); (b) normal or without acute pneumonia on CT images; (c) lack of follow-up CT images; (d) insufficient image quality due to the severe artifacts affecting the image assessment. Consequently, 95 COVID-19 and 415 non-COVID-19 pneumonia patients were recruited and semi-randomly allocated to the training and internal validation cohorts according to the recruitment time. Another 40 patients with viral pneumonia between February 16, 2020 and March 20, 2020 who met the inclusion and exclusion criteria as an independent and new cohort were included to further test the constructed models. Finally, 115 COVID-19 and 435 non-COVID-19 pneumonia patients were enrolled in this study. The workflow of this study was displayed in Fig. 1. Among the non-COVID-19 patients, 128 were confirmed viral infections, 195 mycoplasma infections, 5 chlamydia infections, 3 fungus infections, and 104 co-infections.

### CT imaging acquisition and interpretation

All the patients underwent non-enhanced chest CT examinations for detecting pneumonia in the supine position during end-inspiration. The CT scans were performed with a 64-section multi-detector CT scanner (uCT780, United imaging or Somatom Definition Flash, Siemens Healthineers, or Light Speed VCT, GE Healthcare, or Acuilion, Toshiba Healthcare). The detailed imaging parameters for different scanners were demonstrated in Additional file 1: Appendix S1.

Initial CT images before any treatment were performed by three experienced radiologists in consensus (H.Z., L.H. and J.L., with 9, 11 and 10 years of experience in thoracic imaging, respectively). The disputes between the radiologists were resolved by consulting another experienced radiologist (D.W. or Z.X., with more than 20 years of experience in thoracic imaging, respectively). All of them were blinded to the results of laboratory tests.

The lesion number, distribution, density, extent, and other features were assessed. Lesion number included single or multiple lesions. Distribution included unilateral or bilateral lungs, peripheral or central or both of the peripheral and central sites. Density included pure GGO, GGO with consolidation, and pure consolidation. Other features consisted of reticulation (intralobular/interlobular septal thickening), air bronchogram, lymphadenopathy within the mediastinum or hilus, and pleural effusion. Lymphadenopathy was defined as the size of lymph node more than 10 mm in short-axis diameter.

The extent of pulmonary involvement was estimated using a semi-quantitative scoring system. Each of the 5 lung lobe involvements was scored from 0 to 5 as follows: 0 (0%), 1(< 5%), 2 (5%-25%), 3 (26%-49%), 4 (50%-75%), and 5 (> 75%) [17]. The total CT score was obtained by summing the scores of the five lobes ranging from 0 to 25.

### Image segmentation and radiomics feature extraction

**Fig. 2:**
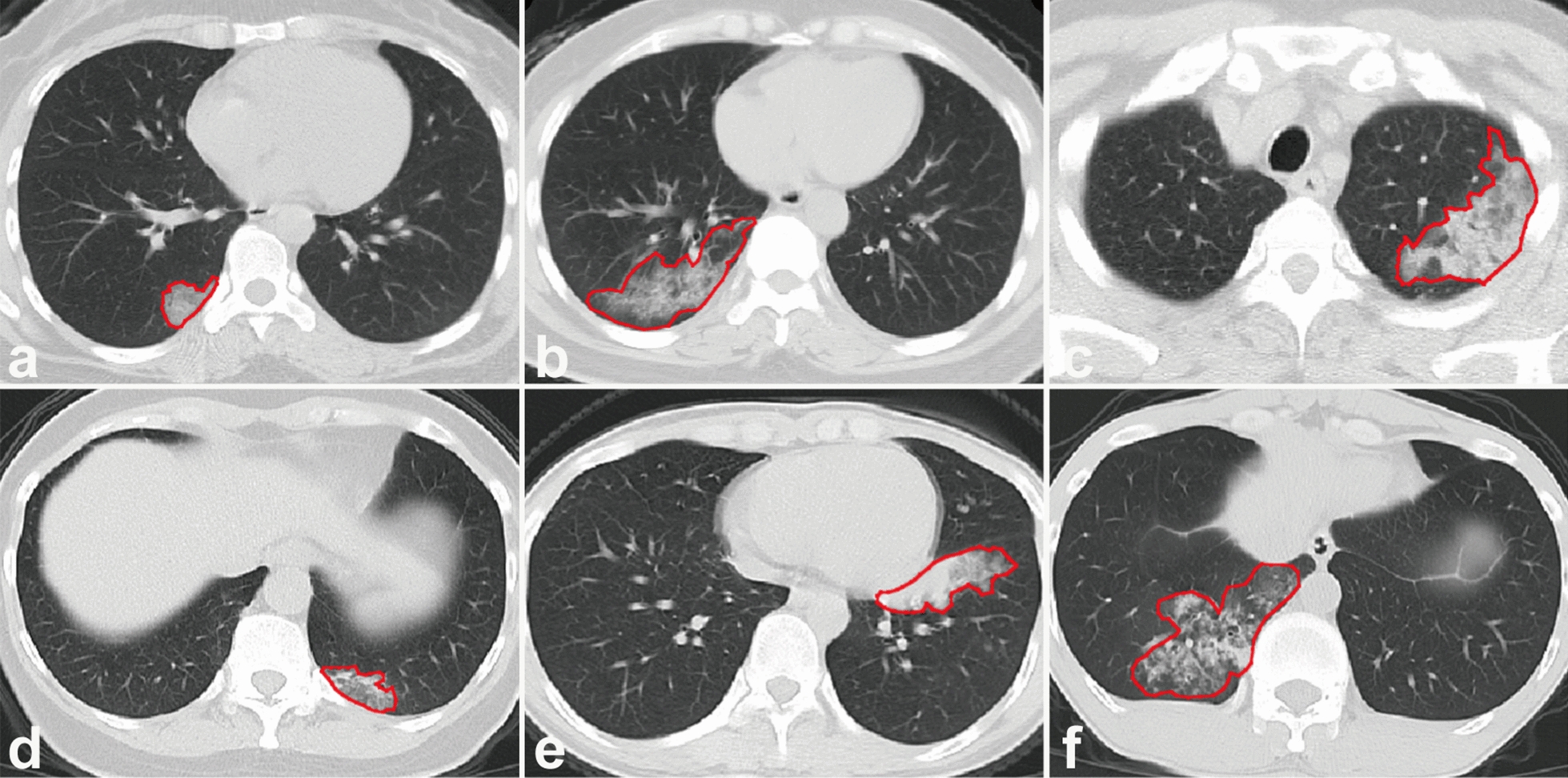
Manual segmentation of 3 COVID-19 pneumonia (**a**–**c**) and 3 non- COVID-19 pneumonia lesions (**d**–**f**)

Three-dimensional (3D) segmentation of the entire volume of interest (VOI) of each pneumonia lesion was performed manually and independently by two experienced radiologists [radiologist 1 (H.R.) and radiologist 2 (R.C.), with 5 and 6 years of experience in thoracic imaging, respectively] via a free and widely used open-source software package (ITK-SNAP, version 3.4.0, www.itksnap.org). The outline of the lesions was delineated along the border on thick-section images with lung window [− 500 Hounsfield unit (HU) level, 1500 HU width] and excluded the large intralesional vessels, bronchi, necrosis, and cavitation (Fig. 2). Both of them were blinded to the results of laboratory tests. VOIs with a volume less than 125 mm^3^ were excluded.

The interobserver and intraobserver reproducibility evaluation of radiomics feature extraction was performed using intraclass correlation coefficients (ICC). Totally 15 VOIs from each group in the training cohort were randomly chosen. The intraobserver ICC was calculated by comparing two segmentations of radiologist 1 (repeated 7-day interval). The interobserver ICC was calculated by comparing segmentation of radiologist 1 (first time) and radiologist 2. An ICC of 0.81 to 1.00 showed almost perfect agreement, 0.61 to 0.80 as substantial agreement, and 0.41 to 0.60 as moderate agreement [14].

Radiomics features were extracted from VOIs by using pyradiomics 3.0.0 version [18] (http://www.radiomics.io/pyradiomics.html). Images were preprocessed and processed using the proposed default setting. During the feature extraction procedure, the CT image was resampled into an isotropic resolution (1 × 1 × 1 mm^3^) to reduce the heterogeneity result from different scanning parameters. We used 25 binwidth to discretize the gray-level intensity to make the calculation of texture features tractable and to process noise-suppressing properties as well. More detailed setting information was described in Additional file 1: Appendix S2. Six classes of radiomics features were extracted: 18 first order statistics features, 14 shape-based features (3D), 22 Gy level cooccurrence matrix (GLCM) features, 16 Gy level run length matrix (GLRLM) features, 16 Gy level size zone matrix (GLSZM) features, and 14 Gy level dependence matrix (GLDM) features. The radiomics feature details were shown in the pyradiomics documentation (https://pyradiomics.readthedocs.io/en/latest/features.html). In addition, two image filters of wavelet and Laplacian of Gaussian were applied to the original image, respectively. Finally, 14 different image types were used for extracting radiomics features.

### Development of clinical and clinico-radiomics combined models

For clinical model, univariate and multivariate logistic regression analysis were applied to select the independent predictors of clinical and radiological features for identifying COVID-19 pneumonia in the training cohort.

For clinico-radiomics model, minimum redundancy and maximum relevance (mRMR), and the least absolute shrinkage and selection operator (LASSO) logistic regression algorithm were used to select the best performed radiomics feature subset in the training cohort. mRMR was performed at first and 30 features were selected, then LASSO was used to select the optimized feature subset with binomial deviation as criterion and obtained the Radscore which was calculated for each lesion by using a linear combination of selected radiomics features and their weighted coefficients. The mean Radscore (mRadscore) of lesions for each patient was used for predicting COVID-19 pneumonia. A clinico-radiomics combined nomogram was developed with the selected clinical variables and Radscore by using multivariate logistic regression analysis.

### Internal validation and clinical utility of clinical and combined radiomics models

The diagnostic performance of clinical and combined models was assessed by using the receiver operating characteristic curve (ROC) analysis, in which the areas under the curve (AUCs), accuracies, sensitivities, and specificities were established. Then, the diagnostic performance of the models was validated in the validation cohort. Calibration curves, obtained by plotting the actual COVID-19 pneumonia probability against developed model-predicted probability of COVID-19 pneumonia, were performed to assess the goodness-of-fit of the clinical and combined models.

Decision curve analysis (DCA) was implemented to evaluate the net benefits of the prediction models at different threshold probabilities in the validation cohort.

### Predictive performance of combined radiomics model in distinguishing COVID-19 from other viral pneumonia compared with clinical model and CO-RADS

Another independent testing cohort including 20 patients with COVID-19 pneumonia and 20 patients with other viral pneumonia was used to test the discriminatory power for the clinical model, clinico-radiomics combined model, and CO-RADS category approach. The CO-RADS included 6 levels of suspicion for pulmonary involvement of COVID-19 besides CO-RADS 0, not interpretable (scan technically insufficient for assigning a score) as follows [11]: CO-RADS 1, very low (normal or non-infectious); CO-RADS 2, low (typical for other infection but not COVID-19); CO-RADS 3, equivocal/unsure (features compatible with COVID-19, but also other diseases); CO-RADS 4, high (suspicious for COVID-19); CO-RADS 5, very high (typical for COVID-19); CO-RADS 6, proven (RT-PCR positive for SARS-CoV-2). The detailed information for each level was demonstrated in Additional file 1: Appendix S3.

The CO-RADS categories for the 40 patients were independently performed by two experienced radiologists who were familiar with the CO-RADS categories and blinded to laboratory results (H.Z. and J.L., with 9 and 10 years of experience in thoracic imaging, respectively). The interobserver agreement was assessed by using Cohen kappa test, where 0–0.2 was slight agreement, 0.21–0.40 fair agreement, 0.41–0.60 moderate agreement, 0.61–0.80 substantial agreement, and 0.81–1.00 almost perfect agreement [19]. The discriminatory power for the three methods was compared.

### Statistical analysis

Quantitative variables were described as mean ± standard deviation or median (inter-quartile range, IQR), as appropriate. The categorical data were expressed as the frequency (percentage). Comparisons of patient characteristics between COVID-19 and non- COVID-19 pneumonia groups were performed by independent two-sample t test, Mann–Whitney U test, and chi-squared test or Fisher’s exact test via SPSS 23.0 (IBM). Other statistical analyses were performed with R software (version 3.6.1, http://www.Rproject.org). Youden’s index was used to determine the optimal threshold that would maximize the sum of sensitivity and specificity for ROC analysis. The AUCs were compared by DeLong test [20]. A two-sided *P* < 0.05 indicated a statistically significant difference.

## Results

### Patient characteristics

The clinical and radiological features of the 550 patients in the training, validation, and testing cohorts were depicted in Tables 1 and 2. For clinical features, there were significant differences for age, cough symptom, white blood cell count, neutrophil ratio, and lymphocyte count in both of the training and validation cohorts. While compared the COVID-19 pneumonia with other viral pneumonia in the testing cohort, only C-reactive protein showed significant difference. For the radiological features, the lesion distribution was significantly different between the COVID-19 and non-COVID-19 groups for all the three cohorts.

**Table 1:** Clinical characteristics of patients with COVID-19 and non-COVID-19 pneumonia in the training, validation, and testing cohorts

Characteristics	Training cohort (n = 379)		*P* value	Validation cohort (n = 131)		*P* value	**Testing cohort (n = 40)**		*P* value
	Non-COVID-19 group (n = 313)	COVID-19 group (n = 66)		Non-COVID-19 group (n = 102)	COVID-19 group (n = 29)		non-COVID-19 group (n = 20)	COVID-19 group (n = 20)	
Age, median (IQR), years	7.0 (26.0)	47.5 (20.5)	< 0.001	30.5 (49.2)	40.0 (20.5)	0.019	56.5 (30.5)	43.0 (13.0)	0.081
Gender (%)			0.006			0.587			0.749
Female	177 (56.5)	25 (37.9)		62 (60.8)	16 (55.2)		9 (45.0)	8 (40.0)	
Male	136 (43.5)	41 (62.1)		40 (39.2)	13 (44.8)		11 (55.0)	12 (60.0)	
Initial symptoms (%)									
Snotty	6 (1.9)	3 (4.5)	0.407	0 (0.0)	1 (3.4)	0.221	0 (0.0)	1 (5.0)	0.500
Sore throat	7 (2.2)	4 (6.1)	0.201	9 (8.8)	6 (20.7)	0.15	0 (0.0)	2 (10.0)	0.468
Cough	292 (93.3)	52 (78.8)	< 0.001	89 (87.3)	17 (58.6)	0.001	15 (75.0)	16 (80.0)	> 0.999
Sputum	123 (39.3)	20 (30.3)	0.171	18 (17.6)	6 (20.7)	0.709	2 (10.0)	5 (25.0)	0.405
Fever	196 (62.6)	48 (72.7)	0.119	65 (63.7)	24 (82.8)	0.053	11 (55.0)	14 (70.0)	0.327
Dyspnoea	3 (1.0)	4 (6.1)	0.022	2 (2.0)	1 (3.4)	0.531	0 (0.0)	1 (5.0)	0.500
Laboratory test^a^ (%)									
White blood cell count	127 (40.6) /17 (5.4)	9 (13.6) /15 (22.7)	< 0.001	22 (21.6)/ 5 (4.9)	0 (0.0)/ 6 (20.7)	0.014	7 (35.0)/ 0 (0.0)	3 (15.0)/ 3 (15.0)	0.098
Neutrophil count	70 (22.4)/ 16 (5.1)	10 (15.2)/ 3 (4.5)	0.402	28 (27.5)/ 2 (2.0)	8 (27.6)/ 1(3.4)	0.989	8 (40.0)/ 0 (0.0)	5 (25.0)/ 0 (0.0)	0.311
Neutrophil ratio	114 (36.4)/ 34 (10.9)	9 (13.6) / 4 (6.1)	< 0.001	43 (42.2)/ 12 (11.8)	5 (17.2)/ 2 (6.9)	0.017	9 (45.0)/ 0 (0.0)	5 (25.0)/ 0 (0.0)	0.185
Lymphocyte count	68 (21.7)/74 (23.6)	2 (3.0)/ 36 (54.5)	< 0.001	5 (4.9)/ 32 (31.4)	0 (0.0)/ 17 (58.6)	0.007	0 (0.0)/ 10 (50.0)	0 (0.0)/ 5 (25.0)	0.102
Lymphocyte ratio	38 (12.1)/ 107 (34.2)	2 (3.0)/ 34 (51.5)	0.009	10 (9.8)/ 43 (42.2)	1 (3.4)/ 15 (51.7)	0.449	0 (0.0)/ 9 (45.0)	0 (0.0)/ 5 (25.0)	0.185
C-reactive protein	165 (52.7)/ 0 (0.0)	43 (65.2)/ 0 (0.0)	0.065	72 (70.6)/ 0 (0.0)	22 (75.9)/ 0 (0.0)	0.578	18 (90.0)/ 0 (0.0)	11 (55.0)/ 0 (0.0)	0.013

*IQR* inter-quartile range

^a^Data are shown in the order of elevated and decreased results for the laboratory tests

**Table 2:** Radiological characteristics of patients with COVID-19 and non-COVID-19 pneumonia in the training, validation, and testing cohorts

Characteristics	Training cohort (n = 379)		*P* value	Validation cohort (n = 131)		*P* value	Testing cohort (n = 40)		*P v*alue
	Non-COVID-19 group (n = 313)	COVID-19 group (n = 66)		Non-COVID-19 group (n = 102)	COVID-19 group (n = 29)		Non-COVID-19 group (n = 20)	COVID-19 group (n = 20)	
Number (%)			< 0.001			0.001			0.025
Single	179 (57.2)	5 (7.6)		64 (62.7)	8 (27.6)		12 (60.0)	5 (25.0)	
Multiple	134 (42.8)	61 (92.4)		38 (37.3)	21 (72.4)		8 (40.0)	15 (75.0)	
Location (%)			0.131			> 0.999			0.339
Unilateral lung	33 (10.5)	3 (4.5)		6 (5.9)	2 (6.9)		4 (20.0)	1 (5.0)	
Bilateral lungs	280 (89.5)	63 (95.5)		96 (94.1)	27 (93.1)		16 (80.0)	19 (95.0)	
Distribution (%)			< 0.001			< 0.001			0.006
Peripheral	48 (15.3)	46 (69.7)		19 (18.6)	15 (51.7)		6 (30.0)	16 (80.0)	
Central	24 (7.7)	1 (1.5)		3 (2.9)	1 (3.4)		1 (5.0)	0 (0.0)	
Peripheral and Central	241 (77.0)	19 (28.8)		80 (78.4)	13 (44.8)		13 (65.0)	4 (20.0)	
Density (%)			0.544			0.469			0.067
Pure GGO	76 (24.3)	13 (19.7)		20 (19.6)	8 (27.6)		11 (55.0)	4 (20.0)	
Pure consolidation	75 (24.0)	14 (21.2)		23 (22.5)	4 (13.8)		3 (15.0)	4 (20.0)	
GGO with consolidation	162 (51.8)	39 (59.1)		59 (57.8)	17 (58.6)		6 (30.0)	12 (60.0)	
Accompanying features (%)									
Reticulation	22 (7.0)	27 (40.9)	< 0.001	13 (12.7)	8 (27.6)	0.102	9 (45.0)	7 (35.0)	0.519
Air bronchogram	168 (53.7)	34 (51.5)	0.749	52 (51.0)	15 (51.7)	0.944	6 (30.0)	9 (45.0)	0.327
Other findings (%)									
Pleural effusion	31 (9.9)	10 (15.2)	0.212	12 (11.8)	2 (6.9)	0.683	1 (5.0)	0 (0.0)	> 0.999
Lymphadenopathy	46 (14.7)	1 (1.5)	0.003	11 (10.8)	2 (6.9)	0.790	2 (10.0)	0 (0.0)	0.468
CT score, median (IQR)	4.0 (3.0)	5.0 (3.0)	< 0.001	3.0 (3.0)	5.0 (4.5)	0.042	2.5 (3.5)	4.5 (4.8)	0.157

*IQR* inter-quartile range

### Features selection and development of clinical and clinico-radiomics models

After univariate and multivariate logistic regression analysis in the training dataset, 8 clinico-radiological features were selected for building the clinical model, including age, gender, neutrophil ratio, lymphocyte count, location (lateral), distribution, reticulation, and CT score.

**Fig. 3:**
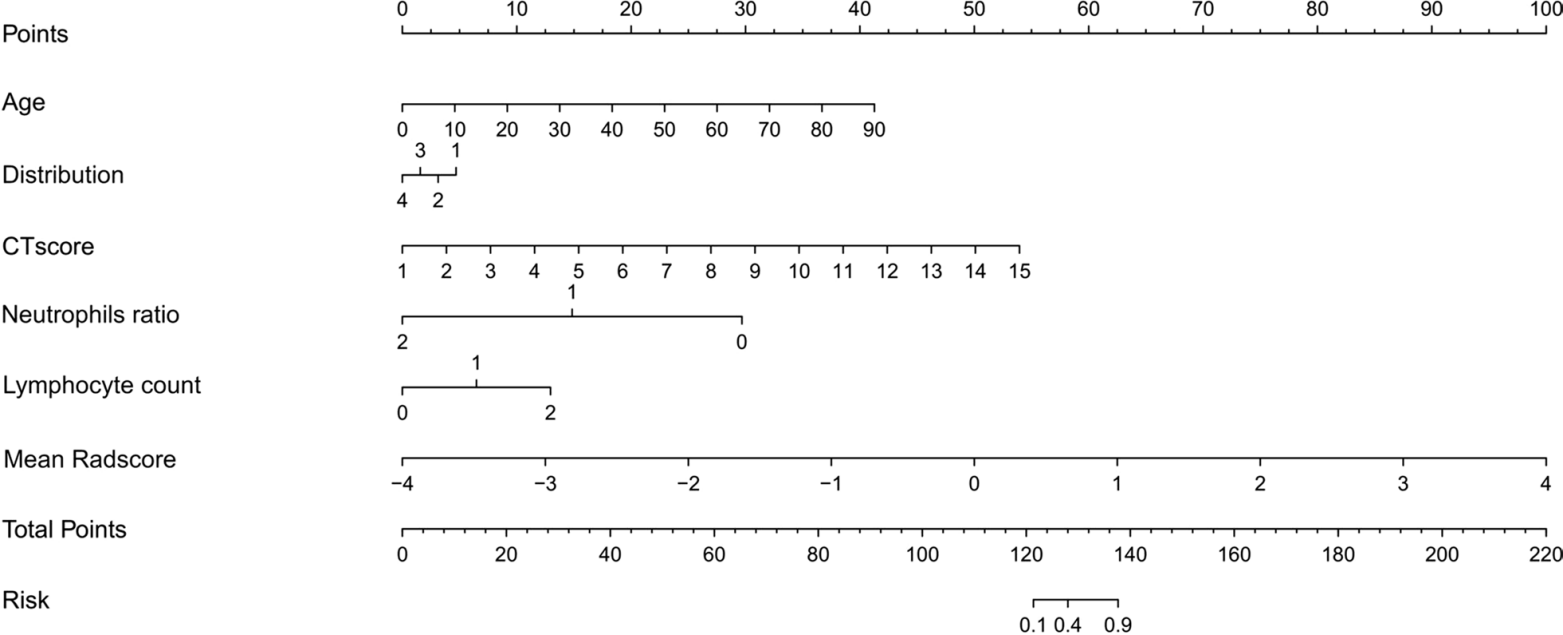
The developed clinico-radiomics nomogram for predicting COVID-19 pneumonia in the training cohort, including 5 clinical features and the mean Radscore

For clinico-radiomics model, a total of 783 lesions in 66 COVID-19 patients and 542 lesions in 313 non-COVID-19 patients were used for extracting radiomics features in the training dataset. Totally 1218 radiomics features were extracted for each lesion. The interobserver and intraobserver reproducibility of radiomics feature extraction was satisfactory with ICCs ranging from 0.7139 to 0.9999, and 0.7130 to 0.9999, respectively. After applying mRMR algorithm and LASSO logistic regression algorithm (Additional file 2: Figure S1), 8 best performed radiomics features were selected to calculate the Radscore including wavelet_LLH_glcm_ InverseVariance, wavelet_HHL_ firstorder_RootMeanSquared, wavelet_LHL_gldm_SmallDependenceHighGray LevelEmphasis, wavelet_LHL_glcm_ClusterProminence, log_sigma_1_0_mm_3D_ glrlm_LongRunLowGrayLevelEmphasis, wavelet_HLL_gldm_LargeDependence LowGrayLevelEmphasis, wavelet_LHL_gldm_LargeDependenceLowGrayLevel Emphasis, and wavelet_HHL_glrlm_LongRunLowGrayLevelEmphasis (Additional file 3: Figure S2). A clinico-radiomics combined nomogram including the mRadscore and 5 selected clinical features was developed (Fig. 3).

### Internal validation and clinical utility of clinical and clinico-radiomics models

**Fig. 4:**
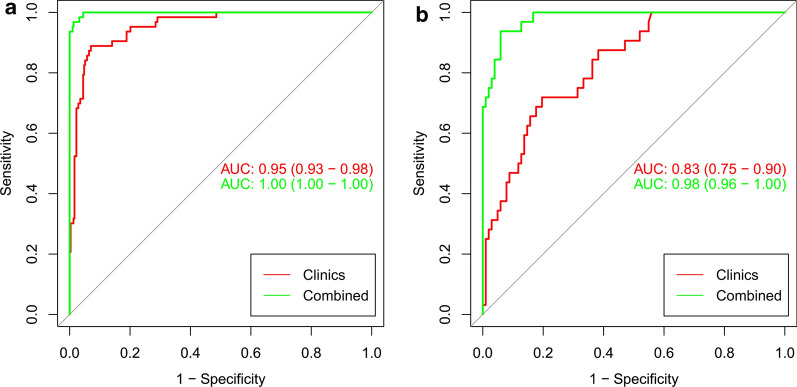
Receiver operating characteristic curve (ROC) analysis for the clinical model and combined radiomics model in the training cohort (**a**) and validation cohort (**b**). The diagnostic performance of the combined radiomics model in distinguishing COVID-19 from pneumonia with other pathogens was better than that of the clinical model in both training and validation cohorts

The AUCs of clinical and clinico-radiomics model developed in the training cohort were 0.95 and 1.00. Favorable performance was observed in the validation cohort. The combined model outperformed clinical model in diagnosing COVID-19 pneumonia, with an AUC of 0.98 compared with 0.83. The sensitivity and specificity of combined model were improved to 0.94 and 0.93. The AUCs, accuracies, sensitivities, and specificities of clinical and combined models in the training and validation cohorts were depicted in Table 3. The ROC analysis results are displayed in Fig. 4. A visual open-source diagnostic tool transformed through the developed clinico-radiomics combined nomogram for diagnosing COVID-19 pneumonia can be achieved through the website (https://duansf.shinyapps.io/COVID-Model/). The detailed representations of the numbers for the clinical variables were demonstrated in Additional file 1: Appendix S4.

**Table 3:** Predictive performance of clinical and clinico-radiomics combined models for diagnosing COVID-19 pneumonia in the training and validation cohorts

	Training cohort		Validation cohort	
	Clinical model	Combined model	Clinical model	Combined model
AUC (95% CI)	0.95 (0.93–0.98)	1.00 (1.00–1.00)	0.83 (0.75–0.90)	0.98 (0.96–1.00)
Accuracy	0.92	0.94	0.79	0.93
Sensitivity	0.89	0.97	0.63	0.94
Specificity	0.93	0.99	0.84	0.93

*AUC* area under the receiver operating characteristic curve, *CI* confidence interval

**Fig. 5:**
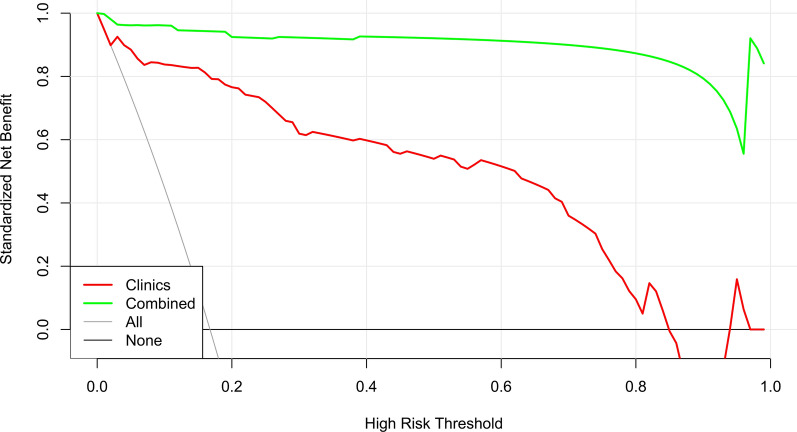
Decision curve analysis for the clinical model and combined radiomics model. The decision curve showed that a combined radiomics model to predict COVID-19 would be more beneficial than the clinical model when the threshold probability was from 0.0 to 1.0

Calibration curves showed that combined radiomics model demonstrated a better agreement between the predicted and actual probabilities of COVID-19 both in the training and internal validation datasets (Additional file 4: Figure S3). DCA revealed that the combined radiomics prediction model was more beneficial than the clinical model, as well as the “treat-all-patients” or “treat-none” strategies when the threshold probability was from 0.0 to 1.0 (Fig. 5).

### Predictive performance of clinical model, clinico-radiomics model, and CO-RADS category in distinguishing COVID-19 from other viral pneumonia

**Fig. 6:**
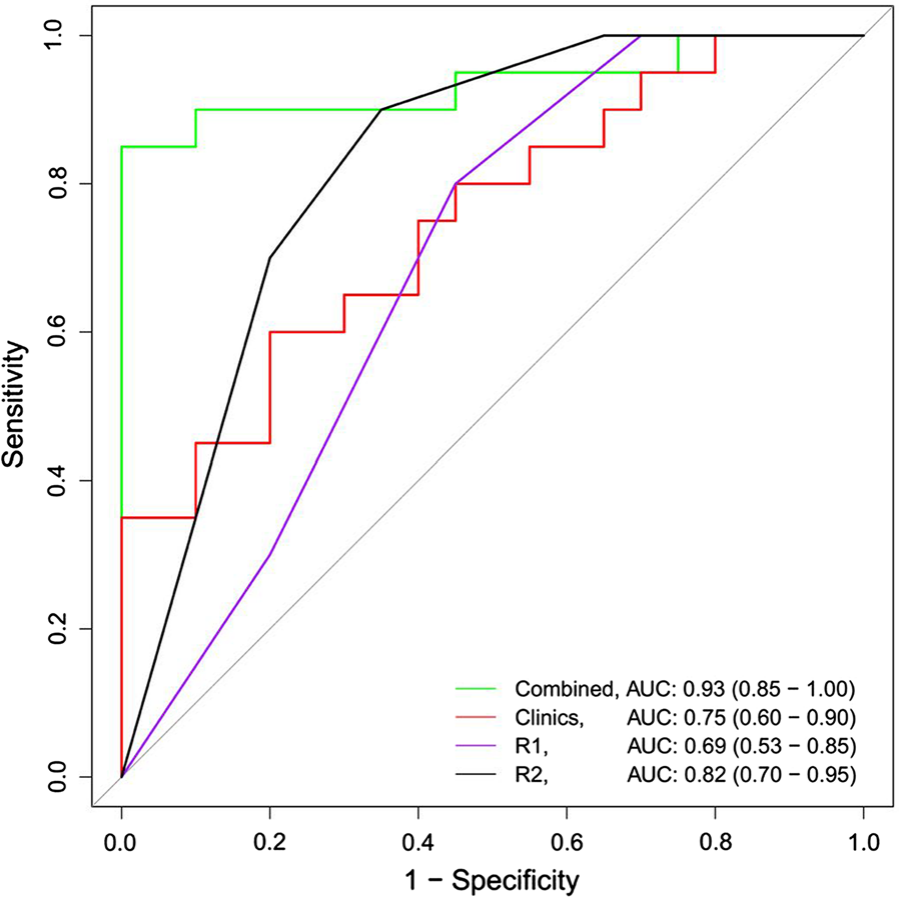
ROC analysis for the clinical model, combined radiomics model, and CO-RADS approach in the testing cohort. The diagnostic performance of the combined model in distinguishing COVID-19 from pneumonia with other viral pneumonia was better than that of the clinical model and CO-RADS approach

In the testing cohort, clinico-radiomics model outperformed clinical model in distinguishing COVID-19 from other viral pneumonia with an AUC of 0.93 compared with 0.75 (*P* = 0.03) (Fig. 6). In addition, the combined model also performed better than two trained radiologists by using CO-RADS. The AUC of radiomics model was higher than 0.69 for radiologist 1 (*P* = 0.008) and 0.82 for radiologist 2 (*P* = 0.15) (Fig. 6). The AUCs, accuracies, sensitivities, and specificities of clinical model, combined model, and CO-RADS in the testing cohort were demonstrated in Table 4. The interobserver agreement between the two radiologists was moderate with a kappa value of 0.53.

**Table 4:** Predictive performance of clinical model, clinico-radiomics combined model, and CO-RADS in distinguishing COVID-19 pneumonia from other viral pneumonia in the testing cohort

	Clinical model	Combined model	CO-RADS	
			Radiologist 1	Radiologist 2
AUC (95% CI)	0.75 (0.60–0.90)	0.93 (0.85–1.00)	0.69 (0.53–0.85)	0.82 (0.70–0.95)
Accuracy	0.65	0.88	0.68	0.78
Sensitivity	0.60	0.85	0.80	0.90
Specificity	0.70	0.90	0.55	0.65

*AUC* area under the receiver operating characteristic curve, *CI* confidence interval, *CO-RADS* COVID-19 reporting and data system

## Discussion

In this study, we developed and validated a combined radiomics model for diagnosing COVID-19 pneumonia, and compared the diagnostic performance with clinical model as well as the performance of two trained radiologists by applying a recently recommended CO-RADS approach. Our results revealed that the combined radiomics model outperformed clinical model in diagnosing COVID-19 pneumonia in the training, validation, and testing cohorts, and not only for the common pathogens’ infection but also for the selective viral infection. The proposed combined model achieved favorable performances with AUC values of 1.00, 0.98, and 0.93 as well as a high sensitivity and specificity in the three cohorts. Furthermore, the combined model was also superior to CO-RADS in discriminating COVID-19 from other viral pneumonia with a sensitivity and specificity of 0.85 and 0.90.

Rapid and accurate diagnosis of COVID-19 is crucial for early intervention and healthcare allocation during the ongoing outbreak. Previous studies had explored the clinical and imaging features of COVID-19 for facilitating the diagnosis of COVID-19 pneumonia, revealing that fever and/or cough, normal or decreased white blood cells, and decreased lymphocyte count, GGO lesions in the peripheral and posterior lungs on CT images could aid in screening the highly suspicious patients [6, 21–23]. However, more common consolidation lesions could be detected due to the time interval from symptom onset and atypical features including fibrous stripes and irregular solid nodules were also presented in the subsequent studies, which complicated the diagnosis [8, 24]. Our study also found that older age, normal neutrophil ratio, decreased lymphocyte count, peripheral distribution on CT as well as higher CT score were independent predictors for distinguishing COVID-19 from non-COVID-19 pneumonia derived from the training cohort, which was in accordance with the above studies. Nevertheless, the predictive performance was not satisfactory with an AUC of 0.83 and a sensitivity of 0.63 in the validation dataset. The various sensitivities and specificities of identifying COVID-19 subjectively with the clinical and radiological features were also found in the previous studies [4, 5, 10].

When evaluating the diagnostic performance of clinical model in discriminating COVID-19 from other viral pneumonia in the testing dataset, the discriminatory power further decreased with an AUC and sensitivity of 0.75 and 0.60. In the previous investigations conducting comparison between chest CT and RT-PCR results, the sensitivity of CT in identifying COVID-19 pneumonia can be estimated to 98%, but the specificity was only 25% by analyzing 1014 patients [4, 5]. Regarding the diagnostic performance among different radiologists from different countries in distinguishing COVID-19 from viral pneumonia on chest CT, the sensitivity, however, was reported to be moderate but the specificity was high [10]. Even by applying the recently recommended CO-RADS approach with reported high discriminatory power of AUC 0.91 in identifying COVID-19 [11], the AUC, sensitivity, and specificity in our study were not satisfactory with 0.69, 0.80, and 0.55, respectively for a trained radiologist familiar with CO-RADS approach, as well as 0.82, 0.90, and 0.65, respectively for the other trained radiologist in distinguishing COVID-19 from other viral pneumonia. The moderate interobserver agreement with a kappa value of 0.53 was also not in favor of the accurate diagnosis of COVID-19. Therefore, it is urgent to develop a more objective approach for improving the current diagnostic accuracy of COVID-19 pneumonia.

Recently, artificial intelligence (AI) using deep learning technology has demonstrated good performance to improve the diagnosis of COVID-19, with sensitivities ranging from 0.67 to 0.97 and specificities from 0.83 to 0.96 [25–28]. With more COVID-19 cases involved, the AI system can achieve more accurate segmentation of COVID-19 pneumonia lesions after training [29]. Additionally, it was reported that the automatic segmentation and classification of AI system would save 30%-40% of detection time for physicians, which is promising in reducing the workload of healthcare system [28]. However, the large amount of data to be trained for deep learning model construction limited its timely application and generalization based on the sporadic COVID-19 cases in most parts of China during the early stage of COVID-19 pandemic. More clinical implementations are warranted for the test of AI system and wide availability. Another machine learning approach radiomics rapidly developed in recent years can be widely available through open-source software and the radiomics signature is easily utilized. The potential for diagnosing and predicting outcomes of different lesions has been proven in the prior reproducible investigations [14, 15], as well as our previous studies in predicting preoperative synchronous distant metastasis in patients with rectal cancer [30, 31]. In this study, 8 radiomics features, mainly focus on the textural features, were selected to build the radiomics signature and the proposed combined radiomics model performed well not only in the training cohort but also in the validation and testing cohorts with AUCs of 1.00, 0.98, and 0.93, respectively. The high sensitivities and specificities with 0.97 and 0.99 in the training cohort as well as 0.94 and 0.93 in the validation cohort were observed.

It was reported that there were overlaps in imaging findings between COVID-19 and other viral infections, such as the coronavirus SARS-CoV and MERS-CoV pneumonia, as well as H1N1, H5N1, influenza, human parainfluenza virus, respiratory syncytial virus, rhinovirus, adenovirus, and so on [6, 23, 32]. Therefore, it is not difficult to understand that the textural features outperformed the other extracted morphological features or the first-order statistical features according to the histogram analysis. Textural features encoded the relationships between nearby voxels within VOIs, reflecting the intralesional heterogeneity. It is the advantage that radiomics can transform conventional medical images into quantitative and high-dimensional data visual analysis [33, 34]. To further test the robustness of the combined radiomics model, we enrolled an independent testing cohort including viral infection patients to assess the diagnostic performance. The AUC, accuracy, sensitivity, and specificity were satisfactory with values of 0.93, 0.88, 0.85, and 0.90, respectively. When compared with the clinical model and the CO-RADS for identifying COVID-19 pneumonia, the AUC value of combined radiomics model was significantly higher. The high sensitivity and specificity can not only facilitate to select the highly suspicious patients of COVID-19 for timely management, but also help to exclude the negative patients for relieving the stress of healthcare system. Different from the current AI systems mainly focusing on the image features, our combined radiomics model incorporated both the independent clinical predictors and radiomics features, which could provide more valuable information for identifying COVID-19 pneumonia. In addition, we further transform the clinico-radiomics nomogram into a visual open-resource diagnostic tool, which is easily used for rapid diagnosis of COVID-19.

Our study has several limitations. First, this was a retrospective study conducted in two centers. Prospective investigation with a larger sample size from more centers will be required to validate our proposed model. Second, since we enrolled the non-COVID pneumonia patients with blood laboratory pathogen-confirmation and pneumonia improvement after treatment by follow-up CT scans, limited bacterial infection cases were available due to the lack of bacterial culture. Third, center II were a general hospital with a strong pediatric medical center, thus many children with mycoplasma infections were included in our study. The median age was demonstrated significantly lower than that of the COVID-19 infection patients, where selection bias may exist. However, our non-COVID-19 pneumonia cases were consecutively enrolled from the real word data in our center, and the children was also proved to be susceptible for COVID-19, which definitely needed rapid and accurate differential diagnosis.

## Conclusion

In summary, our preliminary study demonstrated that chest CT-based combined radiomics model outperformed clinical model and CO-RADS in diagnosing COVID-19 pneumonia. The useful quantitative radiomics signature can facilitate the rapid and more accurate diagnosis as well as timely management of COVID-19.

## Supplementary Information

**Additional file 1.** More detailed information about the imaging parameters of chest CT, radiomics feature extraction, CO-RADS classification, and clinico-radiomics combined model.

**Additional file 2: Figure S1.** Radiomics feature selection using LASSO logistic regression model. (a) The hyper parameter (λ) was selected via ten-fold cross-validation based on minimum criteria. Log (λ) is plotted on the x-axis, and binomial deviance is plotted on the y-axis. The dotted vertical lines indicate optimal values determined using the minimum criteria and one standard error of the minimum criteria (1-SE). log (λ)=-6.71. (b) LASSO coefficient profiles of the radiomics features. Coefficient profiles are plotted against log (λ). The optimal 8 non-zero coefficients were generated at the value selected using ten-fold cross-validation.

**Additional file 3: Figure S2.** The selected radiomics features and their weighted coefficients.

**Additional file 4: Figure S3.** Calibration curves of the clinical and combined radiomics model in the training cohort (a) and validation cohort (b).

## Data Availability

The datasets used and/or analyzed during the current study are available from the corresponding author on reasonable request.

## Abbreviations

COVID-19: Coronavirus disease 2019
SARS-CoV-2: Severe acute respiratory syndrome coronavirus 2
CO-RADS: COVID-19 reporting and data system
RT-PCR: Reverse-transcription polymerase chain reaction
GGO: Ground-glass opacity
ROC: Receiver operating characteristic curve
AUC: Area under the receiver operating characteristic curve
