# Supplementary material for: CT radiomics facilitates more accurate diagnosis of COVID-19 pneumonia: compared with CO-RADS
Source: J Transl Med. 2021 Jan 7;19:29. doi: 10.1186/s12967-020-02692-3 (PMC7790050; doi:10.1186/s12967-020-02692-3)
Supplement: Supplementary file 1 — Additional file 1. More detailed information about the imaging parameters of chest CT, radiomics feature extraction, CO-RADS classification, and clinico-radiomics combined model. [file 12967_2020_2692_MOESM1_ESM.docx]

**Appendix S1. The main parameters for chest CT imaging**

All the patients underwent non-enhanced chest CT examinations for detecting pneumonia in the supine position during end-inspiration. The CT scans were performed with a 64-section multi-detector CT scanner (uCT780, United imaging or Somatom Definition Flash, Siemens Healthineers, or Light Speed VCT, GE Healthcare, or Acuilion, Toshiba Healthcare). The detailed imaging parameters for different scanners were demonstrated as follows.

1. For adults:

tube voltage: 120 kVp, automatic tube current: (120-440) mAs, thickness: (5-7) mm, slice interval: 5 mm, rotation speed: 0.5-1.0 s, helical pitch 1.0875:1 or 1.375:1. Lung window images at 0.625 to 1 mm thickness were reconstructed. Iterative reconstruction technique was implemented.

2. For children:

tube voltage: 100 kVp, automatic tube current: (20-100) mAs, thickness: (3-5) mm, slice interval: (1-3) mm, rotation speed:0.6 s, helical pitch 0·969:1. Lung window images at 0.625 to 1 mm thickness were reconstructed. Iterative reconstruction technique was implemented. The informed consents for CT examination were obtained from all patients.

**Appendix S2. More detailed setting information for radiomics feature extraction by using pyradiomics**

imageType:

Original: {}

LoG:

sigma: [1.0, 2.0, 3.0, 4.0, 5.0] # If you include sigma values >5, remember to also increase the padDistance.

Wavelet: {}

featureClass:

shape:

firstorder:

glcm: # Disable SumAverage by specifying all other GLCM features available

- 'Autocorrelation'

- 'JointAverage'

- 'ClusterProminence'

- 'ClusterShade'

- 'ClusterTendency'

- 'Contrast'

- 'Correlation'

- 'DifferenceAverage'

- 'DifferenceEntropy'

- 'DifferenceVariance'

- 'JointEnergy'

- 'JointEntropy'

- 'Imc1'

- 'Imc2'

- 'Idm'

- 'Idmn'

- 'Id'

- 'Idn'

- 'InverseVariance'

- 'MaximumProbability'

- 'SumEntropy'

- 'SumSquares'

glrlm:

glszm:

gldm:

setting:

interpolator: 'sitkBSpline'

resampledPixelSpacing: [1, 1, 1]

padDistance: 10 # Extra padding for large sigma valued LoG filtered images

binWidth: 25

voxelArrayShift: 1000 # Minimum value in HU is -

label: 1

**Appendix S3. Detailed information about the COVID-19 Reporting and Data System (CO-RADS) classification**

The CO-RADS included 6 levels of suspicion for pulmonary involvement of COVID-19 besides CO-RADS 0, not interpretable (scan technically insufficient for assigning a score) as follows: CO-RADS 1, very low (normal or non-infectious); CO-RADS 2, low (typical for other infection but not COVID-19); CO-RADS 3, equivocal/unsure (features compatible with COVID-19, but also other diseases); CO-RADS 4, high (suspicious for COVID-19); CO-RADS 5, very high (typical for COVID-19); CO-RADS 6, proven (RT-PCR positive for SARS-CoV-2).

**1. CO-RADS 0**

CO-RADS 0 is chosen when scans that are incomplete or of insufficient quality, for example because of severe artifacts due to coughing or breathing.

**2. CO-RADS 1**

CO-RADS 1 implies a very low level of suspicion for pulmonary involvement by COVID-19 based on either a normal CT or CT findings of unequivocal non-infectious etiology. This was modelled on Lung-RADS, where cases with no nodules or with nodules with definitely benign features are reported together. Using the definition, mild or severe emphysema, perifissural nodules, lung tumors, or fibrosis are classified as CO-RADS 1.

**3. CO-RADS 2**

CO-RADS 2 implies a low level of suspicion for pulmonary involvement by COVID-19 based on CT findings in the lungs that are typical of infectious etiology that are considered not compatible with COVID-19. Examples are bronchitis, infectious bronchiolitis, bronchopneumonia, lobar pneumonia, and pulmonary abscess. Features include tree-in-bud sign, a centrilobular nodular pattern, lobar or segmental consolidation, and lung cavitation.

**4. CO-RADS 3**

CO-RADS 3 implies equivocal findings for pulmonary involvement of COVID-19 based on CT features that can also be found in other viral pneumonias or non-infectious etiologies. Findings include perihilar ground-glass, homogenous extensive ground glass with or without sparing of some secondary pulmonary lobules, or ground glass together with smooth interlobular septal thickening with or without pleural effusion in absence of other typical CT findings. CO-RADS 3 also includes small ground glass opacities that are not centrilobular (otherwise CO-RADS 2) or not located close to the visceral pleura (otherwise CO-RADS 4). In addition, it contains patterns of consolidation compatible with organizing pneumonia without other typical findings of COVID-19.

**5. CO-RADS 4**

CO-RADS 4 implies a high level of suspicion for pulmonary involvement by COVID-19 based on CT findings that are typical for COVID-19 but showing some overlap with other (viral) pneumonias. Findings are similar to CO-RADS 5 but are not located in contact with the visceral pleura or are located strictly unilaterally, are in a predominant peribronchovascular distribution, or are superimposed on severe diffuse pre-existing pulmonary abnormalities.

**6. CO-RADS 5**

CO-RADS 5 implies a very high level of suspicion for pulmonary involvement by COVID-19 based on typical CT findings. Mandatory features are ground-glass opacities, with or without consolidations, in lung regions close to visceral pleural surfaces, including the fissures, and a multifocal bilateral distribution. The vicinity to the minor or major fissure is also typical. Subpleural sparing is allowed to be present. CO-RADS 5 requires the presence of at least one confirmatory pattern, which aligns with the temporal evolution of the disease. The crazy paving pattern, which has been described to appear later in the course of the disease, shows visible intralobular lines. As the disease progresses, increasing consolidations occur within the ground-glass areas. Finally, opacities occur that resemble organizing pneumonia, such as reverse halo signs or ground glass with extensive subpleural consolidations and air bronchograms. Subpleural curvilinear bands or bands of ground glass with or without consolidation in a tethered, arching pattern with small connections to the pleura are also considered typical. Thickened vessels within lung abnormalities are typical and frequently found in all other confirmatory patterns. CO-RADS 5 is largely identical to the “typical appearance” of the RSNA consensus statement.

**7. CO-RADS 6**

CO-RADS 6was introduced to indicate proven COVID-19 as signified by a positive RT-PCR test for virus-specific nucleic acid.

**Appendix S4. Detailed representations of the numbers for the clinical variable**

**Distribution:** 1 - 4 represents for the outer one-third of the lung, the middle one-third of the lung, the inner one-third of the lung, and involved more than two-third zones of the lung, respectively.

**Neutrophils ratio**: 0 - 2 represents for normal, elevated, and decreased, respectively.

**Lymphocyte count**: 0 - 2 represents for normal, elevated, and decreased, respectively.
