# Supplementary figures and images for: CT radiomics facilitates more accurate diagnosis of COVID-19 pneumonia: compared with CO-RADS
Source: J Transl Med. 2021 Jan 7;19:29. doi: 10.1186/s12967-020-02692-3 (PMC7790050; doi:10.1186/s12967-020-02692-3)

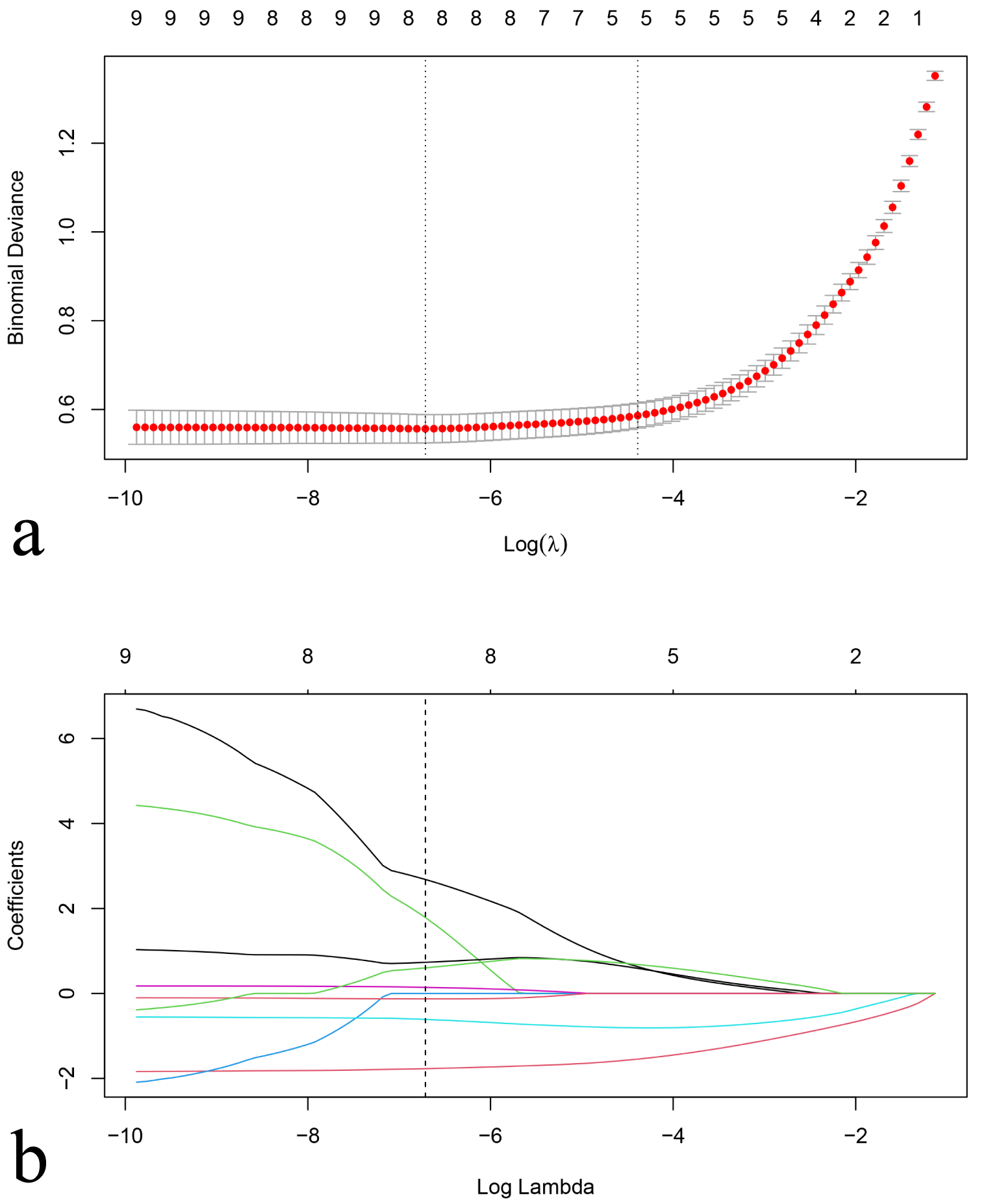

Supplement: Supplementary file 2 — Additional file 2: Figure S1. Radiomics feature selection using LASSO logistic regression model. (a) The hyper parameter (λ) was selected via ten-fold cross-validation based on minimum criteria. Log (λ) is plotted on the x-axis, and binomial deviance is plotted on the y-axis. The dotted vertical lines indicate optimal values determined using the minimum criteria and one standard error of the minimum criteria (1-SE). log (λ)=-6.71. (b) LASSO coefficient profiles of the radiomics features. Coefficient profiles are plotted against log (λ). The optimal 8 non-zero coefficients were generated at the value selected using ten-fold cross-validation. [file 12967_2020_2692_MOESM2_ESM.tif]

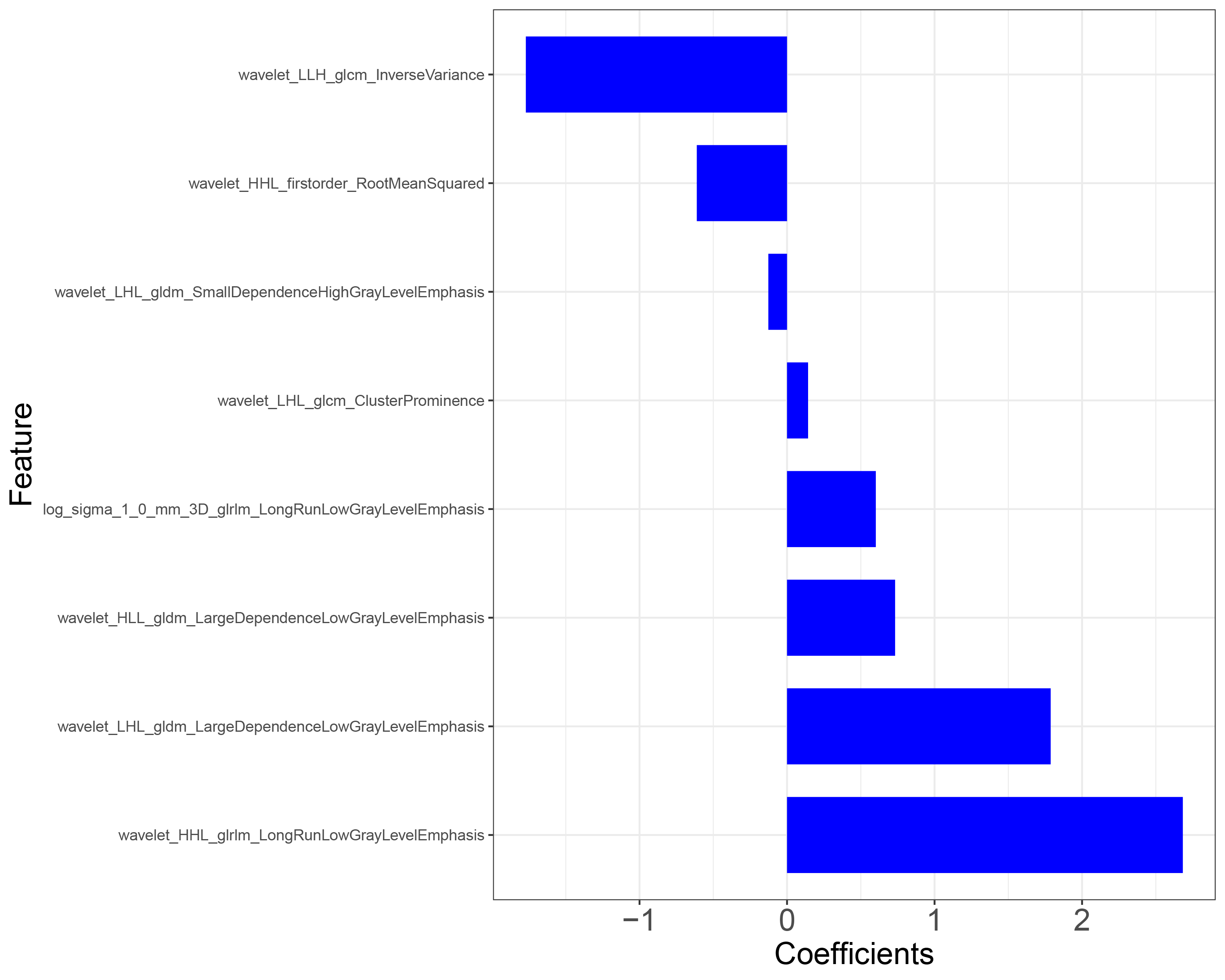

Supplement: Supplementary file 3 — Additional file 3: Figure S2. The selected radiomics features and their weighted coefficients. [file 12967_2020_2692_MOESM3_ESM.tif]

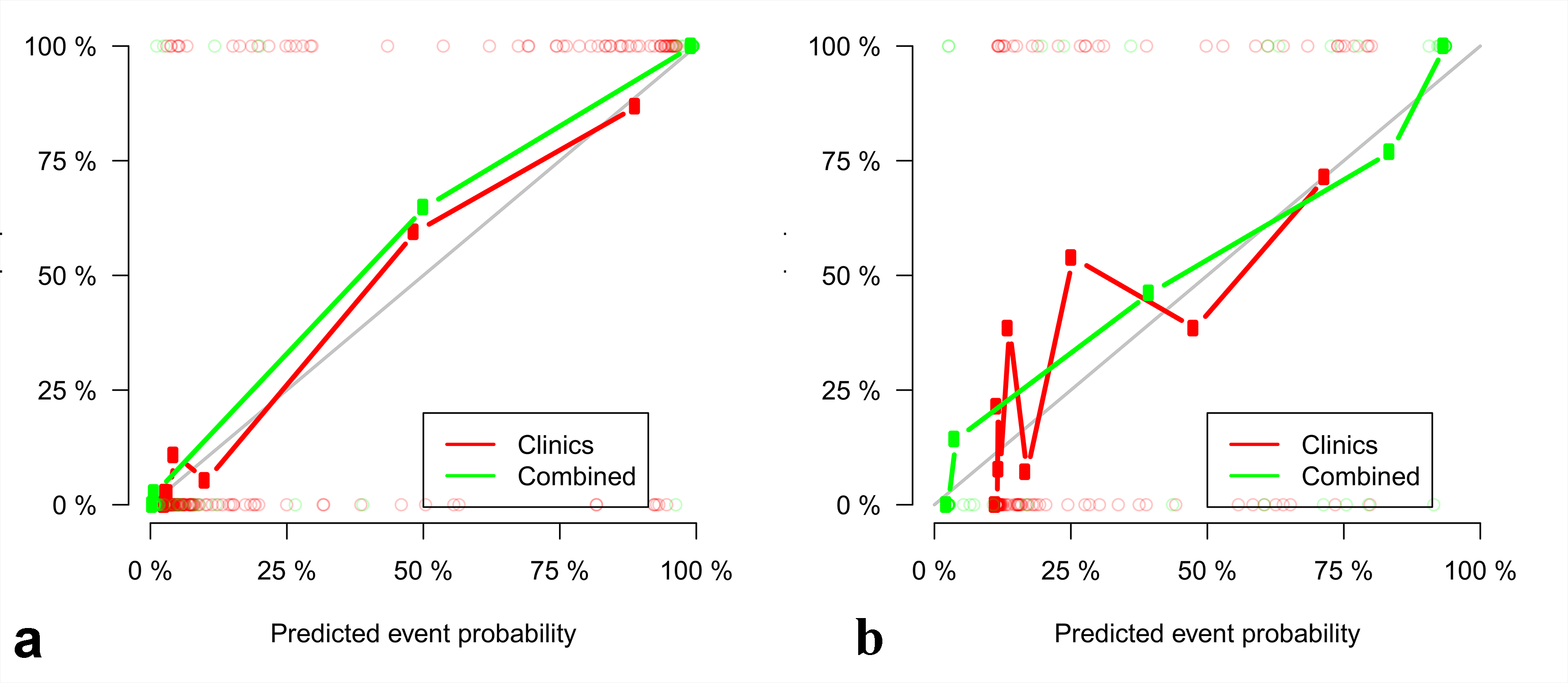

Supplement: Supplementary file 4 — Additional file 4: Figure S3. Calibration curves of the clinical and combined radiomics model in the training cohort (a) and validation cohort (b). [file 12967_2020_2692_MOESM4_ESM.tif]
